# P-688. Pediatric Pneumonia trapped in ‘NET’ (Neutrophil Extracellular Traps)

**DOI:** 10.1093/ofid/ofae631.884

**Published:** 2025-01-29

**Authors:** Barnali Mitra, Debdeep Mitra

**Affiliations:** Command Hospital Air Force Bangalore, Bangalore, Karnataka, India; Assistant Professor, delhi, Delhi, India

## Abstract

**Background:**

Neutrophil extracellular traps (NETs) were initially recognized for their role in the immune system, primarily immobilizing and sometimes killing microorganisms. However, further research has revealed their involvement in various diseases due to the harmful effects of their components on surrounding tissues. The innate immune system's role in pneumonia pathogenesis has been emphasized in several studies. Investigating the involvement of NETs in pulmonary infections and their potential as biomarkers for community-acquired pneumonia (CAP) outcomes has been explored in adult patients. Current data suggests that controlled NET release and clearance are beneficial in pneumonia, but any imbalance in this process could lead to harmful inflammation or increased risk of infection dissemination and sepsis.

However, there is a gap in understanding NET activity in pediatric lung infections. Therefore, this study aimed to assess NET activity in pediatric pneumonia patients and correlate it with blood culture results and viral panels for respiratory infections.

**Methods:**

It was a Cross sectional Observational study at a tertiary care hospital and all children diagnosed to be suffering from pnuemonia. 30 Pediatric pneumonia patients were assessed at the time of presentation and evaluated clinically, radiologically and severity was scored based on Modified respiratory distress assessment instrument score (mRDAI). NET values were checked at the time of presentation and at the time of recovery. These values of NET were correlated with blood culture results, Respiratory viral panel and mRDAI score.

**Results:**

Higher levels of Neutrophil extracellular traps (NETs) correlated with increased disease severity (mRDAI score) and with a poor prognosis and higher hospital stay and increased mortality. There was no statistical significance in the NET score of bacterial and viral pneumonia.

**Conclusion:**

An increase in Neutrophil extracellular traps (NETs) levels is a common occurrence during severe respiratory infections. It is plausible to hypothesize that their migration to the lungs and subsequent activation could worsen tissue damage and contribute to the progression of the disease. NET has been identified as one of the markers linked with poor prognosis and severe pediatric respiratory symptoms.

**Disclosures:**

**All Authors**: No reported disclosures

